# Daily Association Between Sleep and Stressors: Role of Caregiving at Work and Home

**DOI:** 10.1093/geroni/igaa057.1374

**Published:** 2020-12-16

**Authors:** Taylor Harris, Taylor Vigoureux, Soomi Lee

**Affiliations:** University of South Florida, Tampa, Florida, United States

## Abstract

Previous research shows that adults with children experience poor sleep. We know that poor sleep is associated with experiencing more frequent and severe stressors (i.e, subjective feelings of believing his/her life is uncontrollable, unpredictable and overloading) the following day. This study examined whether the sleep—stressor relationship is stronger for individuals with children than those without. Participants were 61 oncology nurses (92% female). Participants completed a background survey that assessed sociodemographic and work characteristics. Using 14 days of ecological momentary assessments, participants reported their sleep characteristics daily upon waking. Three times daily, they also reported whether they experienced any stressors and how severe those stressors were. Multilevel modeling was used to assess whether the sleep—stressor relationship was stronger in nurses with children than those without. After controlling for sociodemographic covariates, poorer sleep quality was associated with more severe stressors. This daily association was moderated by the presence of children (B=-16.89, p<.01); the association was apparent for individuals with children (B=-5.74, p<.05), but not for those without. The daily association for sleep quality and stressor frequency also differed by the presence of children (B=-0.22, p<.01), although the slope for individuals without children did not reach the statistical significance. These findings suggest that individuals with children are at risk for experiencing a stronger linkage between poorer sleep and greater stressor severity. Improving sleep health among adults with children is critical for stress management. Future studies should examine whether age of children or number of children further influences the sleep—stressor relationship.

